# PI3K-AKT-mTOR and NFκB Pathways in Ovarian Cancer: Implications for Targeted Therapeutics

**DOI:** 10.3390/cancers11070949

**Published:** 2019-07-05

**Authors:** Alia Ghoneum, Neveen Said

**Affiliations:** 1 Departments of Cancer Biology, Wake Forest University School of Medicine, Winston Salem, NC 27157, USA; 2 Departments of Cancer Biology, Pathology, and Urology, Wake Forest University School of Medicine, Wake Forest Baptist Comprehensive Cancer Center, Winston Salem, NC 27157, USA

**Keywords:** PI3K-AKT-mTOR, NFκB, ovarian cancer, therapy

## Abstract

Ovarian cancer is the most lethal gynecologic malignancy in the United States, with an estimated 22,530 new cases and 13,980 deaths in 2019. Recent studies have indicated that the phosphoinositol 3 kinase (PI3K)/protein kinase B (AKT)/mammalian target of rapamycin (mTOR), as well as the nuclear factor-κ light chain enhancer of activated B cells (NFκB) pathways are highly mutated and/or hyper-activated in a majority of ovarian cancer patients, and are associated with advanced grade and stage disease and poor prognosis. In this review, we will investigate PI3K/AKT/mTOR and their interconnection with NFκB pathway in ovarian cancer cells.

## 1. Introduction

Ovarian cancer (OvCa) is the fifth most common type of cancer and the primary cause of gynecological cancer death in the United States [1]. At least 70% of patients are diagnosed at advanced stages with poor survival rates [2]. Despite aggressive debulking surgeries and adjuvant or neoadjuvant chemotherapy, recurrence is encountered in 70–80% of patients with poor prognosis and high mortality. In addition, there are limited second line treatment options for resistant or recurrent disease [2,3,4]. Therefore, there is an unmet need to understand the most frequently altered pathways that promote disease progression, recurrence, and chemoresistance. 

**Figure 1:**
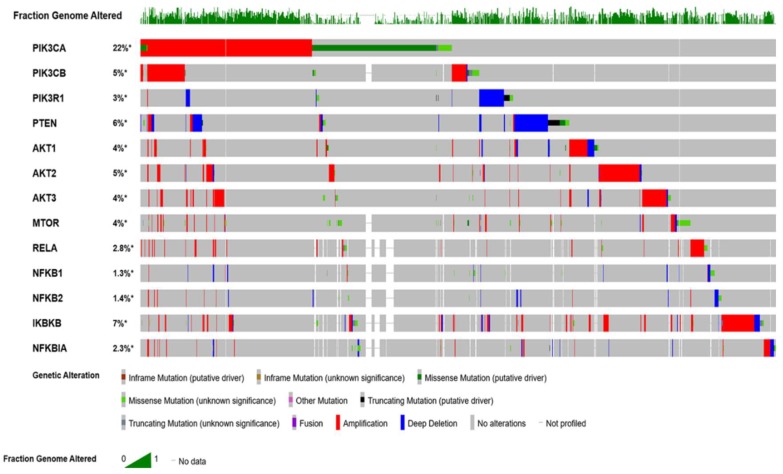
Oncoprint mutation profile of ovarian cancer data curated from The Cancer Genome Atlas (TCGA) datasets. Profile was generated from datasets in cBioPortal (http://www.cbioportal.org) for Cancer Genomics.

The Cancer Genome Altas (TCGA) data revealed the hyperactivation of phosphoinositol 3 kinase (PI3K)/protein kinase B (AKT)/mammalian target of rapamycin (mTOR) (PI3K/AKT/mTOR) pathway in nearly 60% of patients with OvCa [1]. This pathway plays significant multifaceted roles in cancer cell growth, survival, metabolic programing, autophagy, transcription regulation, and angiogenesis [5,6,7]. Patients with high-grade serous cancer (HGSC) have several activating mutations with increased DNA copy numbers of p110α (*PIK3CA*) and p110β (*PIK3CB*) subunits of PI3K [6,8,9]. Other subunits with mutations in OvCa include: PI3K p85 (*PIK3R1*) [10,11], *AKT1* [12], *AKT2* [13], *PTEN* [14], *INPP4B* [15], and *MTOR* [16]. Moreover, the mutation profile of serous ovarian cystadenocarcinoma, generated from cBioPortal for Cancer Genomics (http://www.cbioportal.org/), indicates that several key players in the PI3K/AKT/mTOR/NFκB pathway are hyperactivated (Figure 1).

## 2. Phosphoinositol 3 Kinase (PI3K)

### 2.1. Structural Overview

Phosphoinositol 3 kinase (PI3K) defines a class of lipid kinases that have the ability to phosphorylate the inositol ring 3′-OH group in inositol phospholipids and hence produce phosphatidylinositol (3,4,5)-trisphosphate (PIP3) [17]. The importance of PI3K in signaling is tied to its highly regulated structure. PI3K comprises a family of enzymes divided into: Class I PI3K, which includes catalytic and regulatory subunits. Class IA PI3K includes three isomers (α, β, δ), all of which are activated through receptor tyrosine kinases (RTKs). Class IB includes the group (γ), which is activated via G protein coupled receptors (GPCR) [6,18]. The functions of this family of enzymes are as diverse as their structural derivatives. P110α is a driver of angiogenesis, while p110β, δ, and γ contribute to inflammatory responses. Moreover, p110δ and mTOR play a role in B cell survival during adaptive immunity [18]. Due to its ability to affect several downstream effectors, PI3K Class IA is composed of a regulatory subunit p85 along with the catalytic PI3K 110α subunit [19]. Class II PI3K is divided into three subtypes—PI3KC2α, PI3KC2β, and PI3KC2γ—all of which are catalytic subunits. While both PI3KC2α and PI3KC2β have an N-terminal with a clathrin binding region, PI3KC2α inhibits kinase activity and is implicated in clathrin-mediated endocytosis [20]. The N-terminal of PI3KC2β binds to the scaffold protein intersectin, which is involved in PtdIns(3)P synthesis [21]. Class III PI3K involves two subunits: vacuolar protein sorting 34 (Vps34), the catalytic subunit, and vacuolar protein sorting 15 (Vps15), the regulatory subunit. Vps15 works with Rab5 guanosine triphosphatase (GTPase) to coordinate the activity of Vps34 in endosomal maturation [22].

#### 2.1.1. Interactions of PI3K with Upstream Regulators

**Figure 2:**
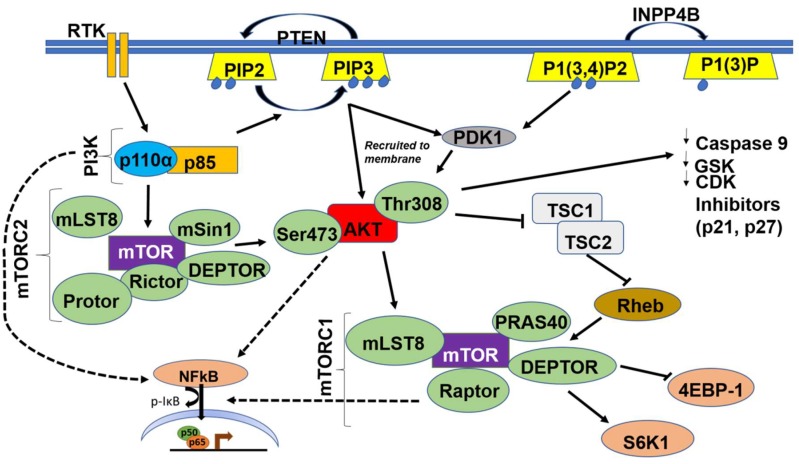
Schematic illustration of the PI3K/AKT/mTOR/NFκB Pathway. Dashed arrows indicate indirect activation of nuclear factor-κ light chain enhancer of activated B cells (NFκB) by PI3K, AKT and mTOR.

Class IA and IB PI3K are activated when an extracellular growth factor or agonist has bound to their cognate receptor tyrosine kinases (RTKs) or to the G-protein coupled receptor (GPCR), respectively [6]. This causes phosphorylation and activation of the regulatory p85 subunit of PI3K followed by Ras activating the catalytic p110α subunit. The activated PI3K heterodimer (PI3K-110α and p85) leads to the conversion of phosphatidylinositol 4,5 bisphosphate (PIP2) to phosphatidylinositol (3,4,5)-trisphosphate (PIP3) [7]. PI3K is also involved in the extracellular matrix (ECM)-integrin-focal adhesion kinase (FAK) and/or integrin-like kinase (ILK) signaling pathway, which is integral for several cellular functions including cellular adhesion, cell cycle progression, cell migration, and invasion [23]. Activated integrins stimulate FAK, a cytoplasmic tyrosine kinase, which in turn activates PI3K [23]; in contrast, ILK is downstream of PI3K and uses the PIP3 generated to activate downstream targets such as AKT and glycogen synthase kinase 3 beta (GSK3β) [24] (Figure 2).

#### 2.1.2. Interactions of PI3K with Downstream Effectors

PI3K triggers the activation of a wide range of downstream effectors via a pleckstrin homology (PH) domain [25]. Protein kinase B (PKB), also known as AKT, is the main effector of PI3K, and serves as a pivotal serine/threonine kinase with multiple downstream targets. Other effectors include kinases, such as Bruton’s tyrosine kinase (BTK), which is important for B-lymphocyte development and differentiation [26]. Effectors also include adaptors, such as GRB-associated binding protein (GAB1/2), tandem PH domain protein 1 (TAPP1), and dual adaptor for phosphotyrosine/3 phosphotyrosine (DAPP), all of which are important for downstream signaling responsible for survival, proliferation, and metastasis. Specific GTPase-activating proteins (GAPs) and guanine nucleotide exchange factors (GEFs) convert guanosine diphosphate (GDP) to GTP which are critical in a cornucopia of cellular processes including cell cycle progression, survival, actin polymerization, cellular polarization, metastasis, and nuclear transport [27,28,29]. PI3K directly activates downstream AKT by docking it to the cellular membrane, and indirectly activates AKT via mammalian target of rapamycin complex 2 (mTORC2) and phosphoinositide-dependent kinase-1 (PDK1), which in turn phosphorylate AKT at the serine 473 and threonine 308 sites, respectively [5,6,30].

#### 2.1.3. The Importance of PI3K 110α in OvCa

In HGSC, PI3K p110α is the most hyper-activated subunit within the PI3K pathway, being altered in nearly 70% of cases [5,31]. Studies have found that mutations in the gene encoding PI3K p110α, *PIK3CA*, are found in nearly 33% of clear-cell carcinoma cases, with the region of 3q26 having increased copy numbers in about 7 out of 9 established OvCa lines [32,33]. *PIK3CA*-activating mutations occur in 20% of endometrioid and clear-cell carcinomas [34]. TCGA data revealed activating mutations and amplifications of *PIK3CA* in 18–28% of cases of serous cystadenocarcinoma, with enrichment of the signature of activated PI3K in a majority of cases irrespective of the subtype [31]. In addition, multivariate survival analysis revealed that PI3K protein expression scores were associated with poor survival in advanced HGSC; in contrast, in univariate analysis, no significant correlation between PI3K protein expression and survival was found [35]. Consistently, data from the Cancer Cell Line Encyclopedia (CCLE) and Domcke et al. [36] revealed activating mutations and amplifications in *PIK3CA* in serous, clear-cell, mucinous, and mixed subtype OvCa cell lines, including cell lines that have been extensively used in preclinical models of OvCa (Table 1). The increased activation of PI3K in OvCa and its role as a hub for several cancer-promoting pathways, explain its many implications in cancer progression including oncogenic transformation, cell proliferation, adhesion, and apoptosis, as well as multiple metabolic pathways [5,7,37,38,39]. PI3K is activated by the over-expressed/hyper-activated receptor tyrosine kinases, such as epidermal growth factor receptors (EGFRs), vascular endothelial growth factor receptors (VEGFRs), and platelet-derived growth factor receptors (PDGFRs) [40], as well as receptors for lysophosphatidic acid (LPA) [41], bioactive lipids, cytokines/ chemokines [42], prostaglandin E2 (PGE2) [43], endothelin [44], and angiotensinogen [45,46,47,48], inducing a plethora of pro-survival, pro-invasive, and pro-inflammatory mediators that further promote OvCa proliferation and invasiveness [41]. Thus, activation of PI3K was shown to be inhibited by RTK and FAK inhibitors, as well as cytokine/chemokine/receptors inhibitors leading to inhibition of OvCa cells migration and invasion through inhibiting downstream PI3K-AKT signaling [49]. 

#### 2.1.4. Correlation of PI3K Mutations in OvCa with Standard of Care Therapy

The vast majority of HGSC patients will experience recurrence with platinum or taxane resistance [50]. Several preclinical studies using cell-based assays indicated that chemo-resistance, specifically to taxanes, is highly correlated with PI3K activation and hyperactivating mutations [51,52]. In OVCAR3, PIK3CA is amplified (six copies) and PIK3R1 contains a splice site on chromosome 5.

**Figure 3:**
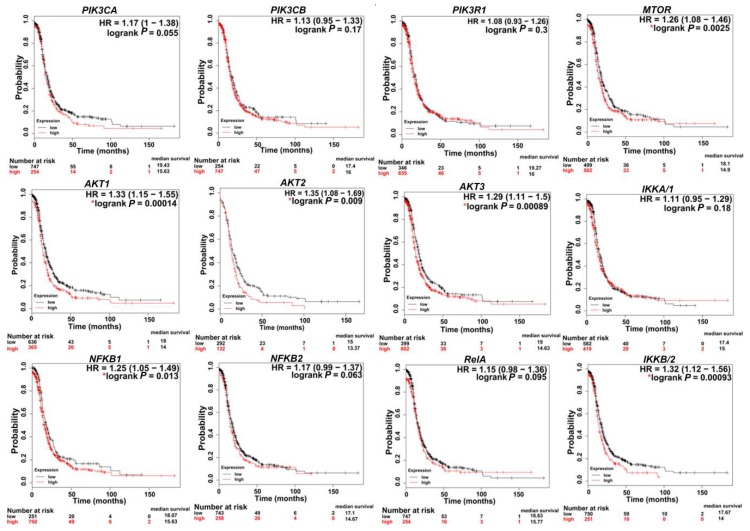
Kaplan–Meier (KM) plots indicating the correlation of PI3K/AKT/mTOR/NFκB expression with patient survival. * *p* < 0.05. KM curves were generated via a KM plot using only JetSet best probe set with analysis restricted to: Serous and Stages 3 + 4.

In SKOV3, PIK3CA contains a missense mutation on chromosome 3. In CaOv3, PIK3CA is amplified with 6.7 copies, and in IGROV1, both PIK3CA and PIK3CB contain missense mutations on chromosome 3 (Table 1). Importantly, the *PIK3CA* mutation most commonly detected in human cancers include OvCa patients’ specimens and OvCa cell lines is the H1047R mutation [53]. Further analysis of several human OvCa cell lines revealed that *PIK3CA* mutations coexisted with *KRAS* and/or *PTEN* mutations indicating the requirement of a secondary defect in a co-regulator of PI3K activity for mutant *PIK3CA* to promote transformation. This was further supported by conditional activation of this mutation in the mouse ovary, showing that *Pik3ca*^H1047R^ alone was capable of inducing premalignant hyperplasia with no tumors of the ovarian surface epithelium. A concomitant *Pik3ca*^H1047R^ mutation plus *PTEN* deletion in the mouse ovary led to the development of ovarian serous adenocarcinomas and granulosa cell tumors. Both mutational events were required for early, robust Akt activation [53]. Pharmacological inhibition of PI3K/mTOR in these mice delayed tumor growth and prolonged survival. Several studies have shown that the rate of mutations in the PI3K pathway, especially in p-AKT and p-p70S6K, including missense mutations and amplifications, is correlated with higher rates of chemoresistance [52,54]. Chemo-sensitization could be achieved via downregulation of PI3K and/or its downstream effectors AKT and mTORC1 [55,56,57]. Consistently, in cell lines and 3D OvCa culture models, cisplatin resistance was overcome via upregulation of the tumor suppressor phosphatase and tensin homolog (*PTEN*) and downregulation of PI3K p110α, as well as the phosphorylated form of AKT (pAKT) [58,59,60,61]. Importantly, TCGA data analysis (http://kmplot.com/analysis) [62] revealed that the high expression of transcripts encoding PI3K subunits are correlated with poor patient survival (Figure 3). Moreover, there is a significant tendency of co-occurrence of PI3K/AKT/MTOR/NFκB mutations and alteration in OvCa (Table 2). While significant evidence exists that implicate mutations in PI3K/AKT/mTOR/NFκB in OvCa aggressiveness, it is important to note that the genomic architecture of OvCa is heterogeneous and multifaceted. PIK3CA mutations are considered driver mutations that provide transformative and positive advantages for cancer cells growing mainly in clear-cell, endometroid, and mucinous OvCa [63]. An integrated study of multi-omics data collected from the TCGA indicated that in HGSC, driver genes included well-known genes such as *CCNE1* (cyclin E1), *CDKN2A* (p16), *KRAS* (Kirsten rat sarcoma), *PTEN*, and *RB1* (retinoblastoma protein 1), as well as more obscure genes such as *EVI2A* (ecotropic viral integration site 2A), *C1orf114* (coiled-coil domain containing 181), and *LCP2* (lymphocyte cytosolic protein 2) [64,65]. Thus, in HGSC, *PTEN* inactivation is considered an activating factor for *PIK3CA.*

## 3. Protein Kinase B PKB/AKT

### 3.1. Structural Overview

The AKT/PKB family encompasses a group of serine threonine kinases, which are cAMP- and cGMP-dependent [66]. The three AKT isoforms—AKT1 (PKBα), AKT2 (PKBβ), and AKT3 (PKBγ)—all contain a conserved N-terminal plekstrin homology (PH) domain, a central kinase domain, and a C-terminal regulatory domain that holds a hydrophobic region. The PH domain binds to the PIP3 at the inner side of the cell membrane and allows for the phosphorylation and activation of AKT [17,67]. The serine and threonine phosphorylation sites vary in each isoform, giving each its own unique role. AKT1 is involved in cellular growth, angiogenesis, and tumor cell invasiveness. AKT2 mediates glucose homeostasis and AKT3 aids in neuronal development and processing. AKT1 and AKT3 together are involved in embryonic development, while AKT1 and AKT2 act to regulate adipogenesis, as well as skin, bone, brain, and muscle development [68,69,70,71,72,73,74].

#### 3.1.1. Upstream AKT Activation

AKT is the main kinase that integrates upstream signals from PI3K and mammalian target of rapamycin complex 2 (mTORC2) with downstream signals to mTORC1 (Figure 2). These signals play a pivotal role in the phosphorylation of downstream substrates and dictate several cellular activities including survival, proliferation, and migration [38,66,70]. AKT is activated via several mechanisms such as recruitment to the membrane by PIP3, phosphorylation at the threonine 308 site by PDK1, and phosphorylation at the serine 473 site by mTORC2. Activated phospho-AKT phosphorylates and activates mTORC1 at serine 248, either directly or indirectly, by inhibiting tuberous sclerosis complex 1 and 2 (TSC1/2) [67,75,76,77]. 

#### 3.1.2. Downstream Effectors of AKT

AKT affects broad downstream targets that induce proliferation and cell cycle progression, apoptosis, and protein synthesis and cell growth [67]. The role of AKT in cell proliferation is mediated via the inhibition of several substrates. AKT phosphorylates GSK-3α at serine 21 and GSK-3β at serine 9, which inhibit GSK-3 kinase activity, thereby halting glycogen synthase, the key enzyme in glycogenesis, which is an important pathway for tumor cell metabolism [78,79]. In turn, GSK-3 phosphorylates several targets, including beta catenin, cyclin D1, and Myc, leading to their polyubiquitination and proteasomal degradation [67]. AKT inhibits Forkhead Box O4 (FOXO4), a transcription factor that induces cyclin D kinase (CDK) inhibitors, p21 (CIP1), and p27 (KIP1) [67]. Furthermore, AKT phosphorylates CDK inhibitors p21 and p27, hence mitigating their inhibitory effect on cyclins D and E, conferring AKT’s integral role in the regulation of cell cycle progression [66,67].

With regard to its anti-apoptotic effects, AKT inhibits several downstream signaling molecules, including caspase 9, while phosphorylating serine 136 of the Bcl2 associated death protein (BAD), leading to the dissociation of Bcl-2 on the mitochondrial membrane and inhibition of apoptosis [66,80]. Moreover, AKT phosphorylates the Forkhead family of transcription factors (FOXO), causing them to bind to 14-3-3 proteins, thus preventing the nuclear translocation of FOXO-1 and FOXO-3, as well as preventing apoptosis in cancer cells [81]. AKT also activates nuclear factor kappa-light-chain-enhancer of activated B cells (NFκB) and cAMP responsive element binding protein (CREB), as well as anti-apoptotic genes such as X-linked inhibitor of apoptosis (XIAP), survivin, and inhibitor of nuclear transcription factor κB (IkB) kinase [9]. In addition, in vitro studies demonstrated that constitutively active AKT allows for activation and nuclear translocation of mouse double minute 2 homolog (MDM2), an E3 ubiquitin protein ligase that degrades p53, reducing cellular p53 levels, and thereby reduces p53 transcriptional activity [82,83]. Moreover, AKT promotes protein synthesis and cell growth by inhibiting TSC2, a tumor suppressor that is responsible for inhibiting cell growth in various cancer types, and 4E-binding protein 1 (4E-BP1), a regulator of mRNA translation and cellular proliferation [7,9,67,84,85].

#### 3.1.3. Inhibition of AKT. 

AKT is inhibited by tumor suppressors including PTEN and inositol polyphosphate 4-phosphatase type II (INPP4B). PTEN dephosphorylates PIP3 to produce PIP2, hence decreasing the downstream activation of phosphoinositide-dependent kinase-1 (PDK1) and phosphorylation of AKT [67,86]. It is important to note that the PTEN/PI3K/AKT pathway is not linear [87]. Transgenic mice with *Pten* deletion/mutation exhibited accelerated tumorigenesis and increased levels of activated AKT [88]. However, in *Pten*-deficient prostate cancer models, deletion of the p110β, but not p110α, subunit of PI3K was able to inhibit tumorigenesis and AKT activation [89], implying the direct effect of p110β-PI3K on AKT activation. These were also reported in patients with various neoplasms including OvCa, endometrial [90], esophageal cancers [91], and prostate cancers, as well as B-cell chronic lymphocytic and acute myeloid leukemia [92,93]. Another mechanism of inactivation of AKT is through INPP4B, which dephosphorylates and inactivates PI 3,4 bisphosphate (P13, 4P2), a direct activator of AKT, thereby obstructing AKT-induced downstream signaling. Loss of function mutations of INPP4B are highly prominent in OvCa [94] and have been correlated with increased ovarian and breast tumorigenicity in xenograft models and across patient samples [67,95,96,97]. 

#### 3.1.4. AKT in OvCa 

In OvCa, activated AKT causes growth deregulation and strong resistance to apoptotic stimuli, leading to uncontrolled tumor growth and cell invasion [7]. AKT is classified as an oncogene when there is a point mutation at the PH domain, and can be activated, independent of upstream PI3K signaling. Specifically, studies have indicated that AKT1 is mutated at E17K and Q79K sites, and AKT2 is amplified in about 40% of OvCa [7,98]. AKT overexpression in OvCa increases their susceptibility to platinum resistance [54]. Ectopic overexpression of AKT in OvCa cell lines increased their cisplatin resistance [99]. Cisplatin-resistant OvCa cell line A2780cis exhibited higher AKT expression levels than its cisplatin sensitive isotype [100]. Similarly, cell lines expressing higher levels of the activated AKT (phosphoAKT) were more resistant to paclitaxel [54,101]. Survival analysis of advanced stage HGSC (stages 3 + 4) in TCGA data using Kaplan–Meier (KM) plot (http://kmplot.com/analysis) [62] indicates that higher expression levels of AKT isoforms is associated with poor patient survival rates (Figure 3). 

## 4. Mammalian Target of Rapamycin (mTOR)

### 4.1. Structural Overview 

mTOR comprises two biochemically and functionally independent catalytic complexes, mTORC1 and mTORC2. The two complexes share two subunits, DEP domain containing mTOR-interacting protein (DEPTOR) and mammalian lethal with SEC18 protein 8 (mLSt8) (Figure 2). Subunits unique to mTORC1 are Raptor and protein rich AKT substrate 40 (PRAS40), and those unique to mTORC2 are protein observed with Rictor-1 (Protor), mammalian stress-activated protein kinase interacting protein 1 (mSin1), and Rictor [102,103,104,105]. Higher expression levels of MTOR correlated with poor survival rates (Figure 3). Moreover, several studies have demonstrated that along with AKT, mTOR is highly hyperactivated and mutated in OvCa, warranting the use of mTOR inhibitors as targeted therapies after standard of care therapies, and in several clinical trials (Table 3) [76,77,106].

#### 4.1.1. Upstream Activation of mTORC1 

Both mTOR complexes are implicated in the induction of angiogenesis, proliferation, and cellular survival [6,103]. For example, during the M-phase of the cell cycle, the active form of mTOR, phospho-mTOR, is expressed at high levels in ovarian granulosa cells, suggesting its instrumental role in cellular checkpoint regulation [107]. mTORC1 plays an important and complex role in nutrient delivery and cellular energy, acting as a cellular energy sensor through AMP-activated protein kinase (AMPK) [102,108]. Once energy levels in the cell decline, AMPK is activated, leading to a downregulation of energy-consuming processes like protein synthesis, and in turn, initiates energy-generating processes such as fatty acid oxidation [108,109]. In addition to the involvement of mTOR with AMPK and cell cycle checkpoints, there is extensive crosstalk between AKT and mTORC1. There are two mechanisms by which AKT activates mTOR. AKT can directly phosphorylate and activate mTORC1, and can indirectly inhibit TSC1/2, activating Ras homolog enriched in brain (RHEB), a GTP-binding protein that activates mTORC1 [77,103]. Moreover, AMPK has been shown to inhibit mTORC1 via two mechanisms, by phosphorylating and activating TSC2, or by phosphorylating RAPTOR and disrupting the mTORC1 complex configuration. Several studies have suggested that PI3K class III member, VPS34, has a regulatory interaction with mTORC1. The inhibitory effect of AMPK on Raptor and the effect of VPS34 on mTORC1 lead to downstream autophagy in malignant cells [102,110,111,112]. 

#### 4.1.2. Downstream Effectors of mTORC1 

Phospho-mTOR activates two downstream targets: the 4E-binding protein 1 (4E-BP1) and ribosomal protein S6 kinase (S6K). Upon stimulation, 4E-BP1 dissociates from eukaryotic translation initiation factor 4E (eIF4E), leading to the subsequent formation of the eIF4F complex and translation initiation [113]. 4EBP1 influences the metabolic components of the tumor microenvironment by regulating specific mRNA translational events [113,114,115,116]. Specifically, in aggressive cancers, 4E-BP1 functions as a hypoxia-inducible switch, allowing for translation of factors, such as VEGF, HIF1α, and Bcl2, and hence facilitating angiogenesis and anti-apoptotic cell growth [85,117]. Phosphorylated S6K is required for cell growth and G1 cell cycle progression [102,109]. The activated mTOR also causes selective translation of proteins involved in survival, including survivin, myeloid leukemia sequence 1 (McL1), and X-linked inhibitor of apoptosis (XIAP); angiogenic factors, such as vascular endothelial growth factor A (VEGF-A) and fibroblast growth factor 2 (FGF2); and DNA repair genes, such as breast cancer 1 (BRCA1), p53 binding protein 1 (53BP1), and H2A histone family member X (γH2AX) [37,75,76,77]. 

#### 4.1.3. Upstream Regulators of mTORC2 

While the upstream regulators of mTORC1 have been established over several studies, regulators for mTORC2 are less clearly defined. Several hypotheses have been proposed to explain mTORC2 regulation. Liu et al. [118] proposed a model whereby stress-activated protein kinase (SAPK)-interacting protein 1 (SIN1) binds and inactivates mTORC2 enabling PIP3 to free mTORC2 from SIN1, and thus, allowing for AKT phosphorylation. A second model proposes a positive feedback mechanism between AKT and mTORC2 whereby AKT phosphorylates SIN1, leading to activation of mTORC2 kinase. mTORC2 then phosphorylates AKT, catalyzing the full activation of AKT [119]. The third hypothesis implies that the TSC complex (TSC1/2) physically associates with and activates mTORC2 in a GTPase-independent manner [120,121]. These hypotheses all suggest that upstream molecules exert a highly complex regulation of mTORC2 signaling.

#### 4.1.4. Downstream Effectors of mTORC2 

The Rictor-mTOR complex forms a hydrophobic motif kinase that phosphorylates and activates AKT on the ser473 [122]. While both Rictor and Sin1 act in conjunction with mTOR, they can also act independently of the mTORC2 complex [102]. Rictor can independently activate ILK, mysosin 1C (Myo1c), and cullin1 (CUL1); while Sin1 can activate c-Jun N-terminal kinase (JNK), Ras, and MAPK/ERK kinase kinase (MEKK2/3), suggesting that Rictor and Sin1 facilitate other cellular activities not dictated by mTORC2 [122,123,124].

#### 4.1.5. mTOR in OvCa

mTOR1 is activated in 55% of epithelial OvCa and this hyper-activated mTOR is involved in cell growth, angiogenesis, and evasion of cell death [31,37,125]. Several studies have reported that nearly 41% and 26.4% of HGSC express the phosphorylated form of mTOR1 targets 4EBP1 and 70S6K, respectively, implying that mTORC1 activity is associated with a more aggressive phenotype and poor prognosis [126]. Consistently, analysis of TCGA data indicated that high expression of MTOR is associated with poor survival rates in patients with advanced stage (stage 3 + 4) HGSC (Figure 3). 

## 5. NFκB

### 5.1. Structural Overview

Nuclear factor-κ light chain enhancer of activated B cells (NFκB) comprises a group of transcription factors that are divided into two classes: Class I, which include NFκB1 or p50/p105 (in which p50 is the processed product of the precursor, p105), and NFκB2 or p52/p100 (in which p52 is the processed product of p100). Class II includes RelA/p65, RelB, and c-Rel. All members have a Rel homology domain that includes the N-terminal and C-terminal domains positioned before the nuclear localization signal. In addition to the Rel homology region, members of Class II in particular, also carry a transactivation domain at the C-terminus [127,128].

#### 5.1.1. Canonical Pathway 

The NFκB canonical pathway includes NFκB1 (a complex composed of the cleaved subunit p50), IKBα, and RelA/p65. IkBα is phosphorylated by the Inhibitor Of Nuclear Factor Kappa B Kinase (IKK) complex at two N-terminal sites: ser32 and er36 [129,130]. The IKK complex is composed of IKKα, IKKβ, and IKKγ (NF-kappa-B essential modulator, NEMO). Upon phosphorylation of NFκB1, the IKBα subunit undergoes ubiquitination and subsequent proteasomal degradation. This allows p50 and RelA to dimerize and translocate to the nucleus where they induce the transcription of genes involved in inflammation, cell growth, and apoptosis. Several factors, such as substrates for antigen receptors and cytokines, can trigger activation of the canonical pathway. In addition to p50 and RelA heterodimer, p50 and c-Rel can also dimerize and enter the nucleus to initiate downstream transcription of antiapoptotic genes, such as Bcl-xL, and genes involved in checkpoint inhibition of the cell cycle [131,132]. 

#### 5.1.2. Non-Canonical Pathway

In this pathway, NFκB2 is composed of p100ANK/RDH and RelB. Activation of this pathway is achieved when inflammatory cytokines, TNF and IL1, bind to their cognate receptors and subsequently signal to NFκB inducing kinase (NIK) to activate the IKK complex [133]. In this case, the IKK complex is solely composed of IKKα dimers [134]. This IKKα homodimer phosphorylates two sites on NFκB2 at serine 866 and 870, leading to the degradation of p100 into p52 [132], dimerization of p52 and RelB subunits, and their translocation to the nucleus, where they induce the transcription of genes involved in immune function specifically of B cells [132].

#### 5.1.3. Regulation of NFκB 

In addition to the regulation of the canonical NFκB pathway by the IkB/IKK complex in the cytoplasm, this pathway is regulated at multiple levels involving heterodimerization, nuclear translocation, and even after p65RelA/p50 heterodimers translocate to the nucleus. In addition, acetyltransferases in the nucleus, specifically p300/CBP, are critical in the acetylation of RelA, mainly at lysines 218, 221, 310, and 314 [135]. Lysine acetylation of RelA influence NFκB DNA binding affinity and transcriptional activation [135,136]. IKBα acts as a post-induction repressor of NF-kappaB/Rel proteins [137,138] as it contains ankyrin repeats that aid in its nuclear localization and attachment to the deacetylated form of p65/RelA, leading to the nuclear export of the NFκB [139]. Canonical NFκB activation can also be occur in an atypical manner by DNA damage and involves formation of a complex between Ataxia Telangiectasia Mutated (ATM), NEMO, and IKKs. RelA interacts through the Rel homology domain with Breast Cancer 1 (BRCA1) to enhance the transcriptional activation of NFκB target genes. This process is dependent upon the phosphorylation of p65/RelA at serine 276, and BRCA1 (Really Interesting New Gene (RING) finger domain [140]. BRCA1 was identified as a regulator of NFκB (p65/p50) activation in response to DNA-damaging agents, etoposide and camptothecin [141]. BRCA1 activates NFκB by facilitating the p65-independent recruitment of the p50 onto the promoter of responsive genes. This activation of NFκB has been shown to contribute to chemoresistance through repression of BRCA1-induced apoptosis in response to DNA damage [141].

#### 5.1.4. PI3K-AKT-mTOR-NFκB Axis

**Figure 4:**
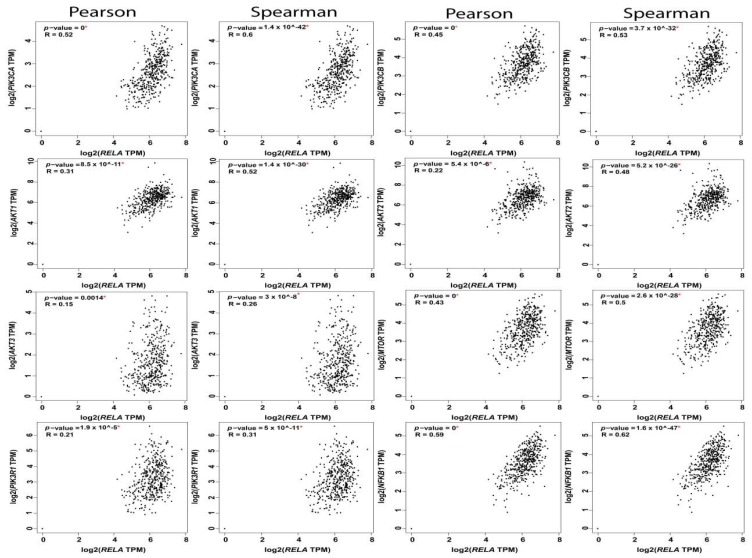
Correlation between RelA and the nodes of PI3K-AKT-mTOR and NFkB pathways.

**Figure 5:**
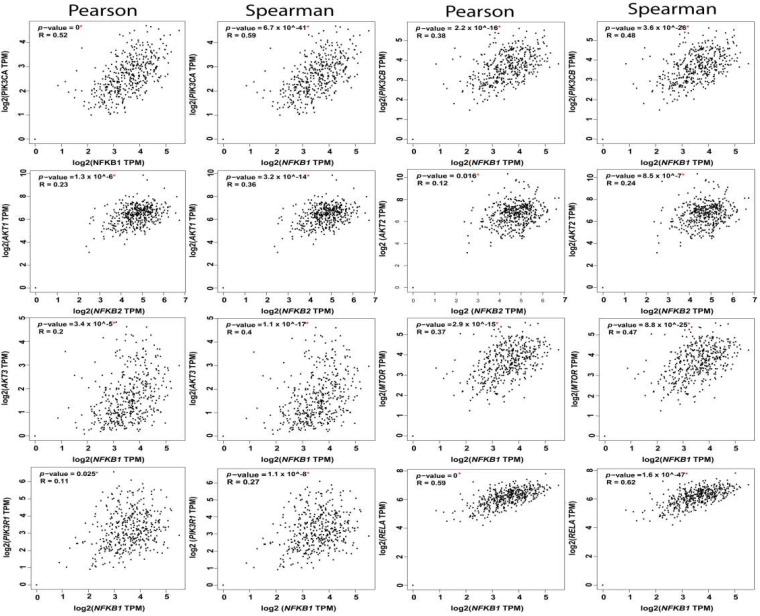
Correlation between NFkB1 subunit and the nodes of PI3K-AKT-mTOR and NFkB pathways.

**Figure 6:**
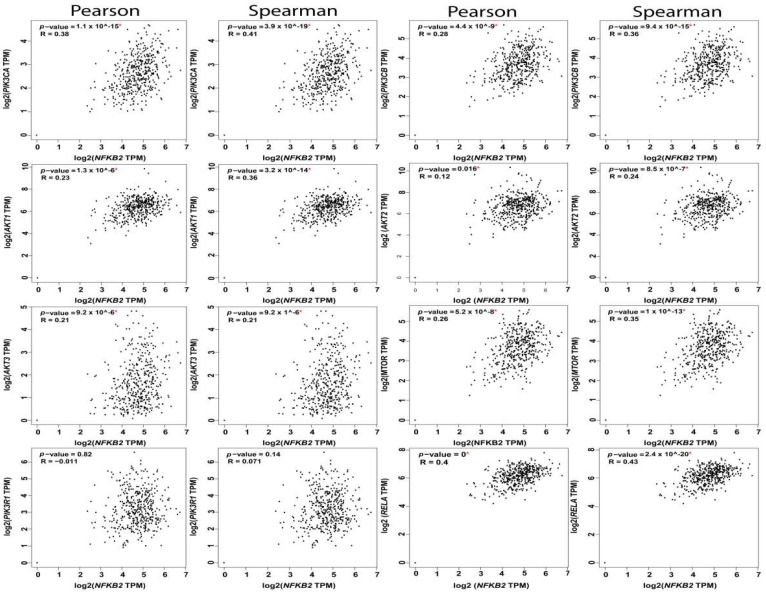
Correlation between NFkB2 subunit and the nodes of PI3K-AKT-mTOR and NFkB pathways.

The reciprocal interactions between the multiple nodes of PI3K-AKT-mTOR and NFκB pathways have been highlighted in myriad physiologic and pathologic contexts. In silico analysis of PI3K-AKT-mTOR and NFkB expression, and correlation in OvCa TCGA samples using KM plot (http://kmplot.com/analysis) [62] and GEPIA web tools (http://gepia.cancer-pku.cn/index.html) [2], revealed that they are not only associated with poor survival, but they also exhibited significant positive correlation at multiple nodes by both Pearson correlation, which determines the linear relationship between two continuous variables, used in combination with the Spearman correlation coefficient, which is chiefly based on ranked values for each variable (Figure 4, Figure 5 and Figure 6).

The PI3K catalytic subunit PI3K-p110α and its regulatory subunit p85 have been shown to directly activate NFκB [142,143,144]. Overexpression of the p110α subunit induced p65RelA activation and nuclear translocation. Consistently, PI3K inhibitors, LY294002, and wortmannin, as well as the dominant negative regulatory p85 subunit, inhibited NFκB activation [142]. PI3K/NFκB interaction is induced via a multi-step cascade triggered by the release of pro-inflammatory cytokines [142,143,144], followed by the interaction between cytokine receptors in the cytoplasm with the regulatory p85 subunit of PI3K, which in turn increases PI3K 110α catalytic activity. Consequently, activated PI3K 110α phosphorylates downstream p65RelA of NFκB followed by its nuclear translocation [142,143,144]. PI3K activation also phosphorylates AKT with subsequent activation of the p65RelA subunit of NFκB via phosphorylation at S534 through the IKK complex. Phospho-AKT mediates the phosphorylation of IKKα at Thr23, allowing for IKKα to phosphorylate IkB, labelling it for polyubiquitination and proteasomal degradation and phosphorylate p65RelA at S534, and hence allowing NFκB to translocate into the nucleus [145]. Furthermore, the activation of the Ras-PI3K-AKT cascade and the p38 mitogen-activated protein kinase induced NFkB activation through binding of its co-activator cAMP responsive element binding protein (CREB) [146]. Moreover, AKT can activate NFkB independent of IKK by directly phosphorylating the transactivation domain (TAD1) of the p65RelA subunit [146]. 

The complex crosstalk between the PI3K pathway and NFκB results in decreased survival rates in OvCa patients [147]. High expression levels of the p65 RelA subunit of NFκB, along with cleaved caspase 3, conferred poor outcomes in OvCa patients [148]. NFκB expression levels were strongly associated with chemoresistance. Mabuchi et al. [149] demonstrated that inhibition of NFκB resulted in enhanced efficacy of cisplatin in in vitro and in vivo OvCa models. Upregulation of p65 RelA subunit of NFκB increased the resistance of OvCa to carboplatin [149], and significantly enhanced the aggressiveness of OvCa cells [150]. The ability of NFκB to influence OvCa chemoresistance was attributed, in part, to its interactions with Homeobox (HOX) transcript antisense RNA (HOTAIR), a long non-coding RNA (lncRNA) that not only cooperated with NFκB to induce platinum resistance, but also decreased IkBα, allowing for constitutive activation of NFκB, suggesting a positive feedback loop during the DNA damage response that enhances cellular senescence and chemoresistance in OvCa [151].

## 6. mTORC1 and NFκB Activation

mTOR downstream from AKT controls NFκB activity in *PTEN*-null/inactive cancer cells via interaction with and stimulation of IKK. The mTOR-associated protein Raptor is required for the ability of AKT to induce NFκB activity. This was further supported by inhibiting IKK activity in PTEN-deficient prostate cancer cells by the mTOR inhibitor rapamycin through a mechanism that may involve dissociation of Raptor from mTOR [152]. Paradoxically, IKKα has been shown to regulate mTORC1 activity in *PTEN*-deficient cancer cells [153,154]. In contrast, IKKβ activates mTORC1 in an AKT-dependent manner [152], and in an AKT-independent manner through the phosphorylation of the mTOR repressor TSC1 at S487 and S511 [155]. The regulation of mTORC1 by IKKβ suggests a reciprocal activation of IKKβ by mTORC1, leading to activation of a NFκB canonical pathway [152,156].

## 7. Therapeutic Targeting of PI3K-AKT-mTOR-NFκB in Preclinical Models of OvCa

The PI3K-AKT-mTOR-NFκB pathway is responsible not only for the aggressiveness of OvCa, but also contributes to the increased resistance to the standard chemotherapeutics, cisplatin and paclitaxel [157]. Recent decades have witnessed an unparalleled expansion of therapeutic agents designed to target the PI3K/AKT/mTOR/NFκB pathway. 

Several preclinical studies indicate the effectiveness of PI3K inhibitors in OvCa, either alone or in combination with other therapies. For example, isoform-specific p110β inhibitors have demonstrated a strong potency, especially against *PTEN*-deficient cell lines [158]. While some preclinical data suggests that OvCa cells with simultaneous mutations in KRAS and loss of PTEN depend more heavily on p110α than p110β [159], concurrent inhibition of p110α and p110β may yield optimal tumoricidal activity in OvCa [160]. Both p110α and p110β isoforms exhibit overlapping roles and the success of p110β specific inhibitors likely depends on the absence of p110α-activating mutations [161]. A combination of PARP inhibitor, Olaparib, and PI3K inhibitor, BKM120, effectively blocked the proliferation of OvCa cells with an intensified DNA damage response blockade in vitro, and synergistically inhibited the growth of *PIK3CA* mutant OvCa cells SKOV3, IGROV1, and HEYA8 cells. Additionally, BKM120 synergized with Olaparib to increase apoptosis as well as inhibit migration and invasion of PIK3CA mutant OvCa cells [162]. Interestingly, OvCa cells that responded favorably to the combination treatment demonstrated decreased BRCA1/2 expression and decreased intraperitoneal dissemination [162]. Combining the DNA-damaging drug, doxorubicin, with a class I PI3K inhibitor, GDC-0941, in OvCa preclinical studies showed that PI3K inhibition significantly increased apoptosis, DNA damage, and the overall anti-tumorigenic effects of doxorubicin [163]. 

AKT protein expression was significantly increased in cisplatin-resistant A2780 OvCa cells (A2780cis) as compared to its cisplatin-sensitive counterpart [99,100]. This platinum resistance in the A2780cis cell line was overcome by AKT downregulation by siRNA [99]. The polyadenylation inhibitor cordycepin, also known as 3′-deoxyadenosine [164,165], has been shown to induce apoptosis and inhibit OvCa cell migration through inhibition of CCL5-induced AKT-NFκB signaling pathway [165]. Consistently, Aurora-A inhibitor, MK-5108, induced cell cycle arrest by inhibiting the p65 subunit of NFκB and led to the nuclear accumulation of IκBα [166]. With decreased interleukin-6 (IL-6), interleukin-8 (IL-8), macrophage chemoattractant protein-1 (MCP-1), and Growth-Regulated alpha (GRO-α). Therefore, inhibition of the NFκB pathway demonstrated anti-tumorigenic effects on OvCa stem cells.

Inhibition of the PI3K-NFκB axis also inhibited OvCa stem cells (CSCs) triggered by cisplatin. Disruption of the PI3K-NFκB loop by wortamannin reduced the OvCa CSC subpopulation [167]. A study by Gonzalez-Torres et al. [168] reported that in the SKOV3 cell line, CD44+ cells exhibited increased transcripts of stemness genes *NANOG*, octamer-binding transcription factor 4/3 (*OCT4/3*), and sex determing region Y HMG-box 2 (*SOX2*) with high expression levels of RelA, RelB, and IKKα. Inhibition of the NFkB canonical pathway by dominant negative IkBα, as well as inhibition of non-canonical kinase, IKKα, by shRNAs, decreased CSC population with downregulation of stemness genes [168]. Additional studies by House et al., [169] showed that NFκB promotes OvCa tumor initiating cells in two arms: first, via the canonical pathway that supports proliferative cancer cells, and second, via the non-canonical pathway that supports ALDH+ OvCa stem cell like cells (CSLCs). Another study demonstrated that CSLCs produce CCL5, which activates downstream NFκB signaling and allows for the differentiation of CSLCs to endothelial cells (ECs) that participate in tumor angiogenesis and further promote invasion and metastasis of OvCa cells [170]. 

Preclinical studies of an mTOR inhibitor revealed that CC223 disrupted the assembly of mTORC1-Raptor and mTORC2-Rictor complexes in SKOV3 cells [171]. CC223 was earlier found to inhibit the pro-survival and anti-apoptotic effects of sphingosine kinase 1 (SphK1), which is over-expressed in OvCa cells [172,173]. CC223 also blocked phosphorylation of S6K1 and AKT (Ser473), both of which are indicators of mTOR activity. Moreover, mTOR-dependent proteins, cyclin D1 and hypoxia-inducible factor-1α (HIF1α), were also decreased upon CC223 treatment together with inhibition of cell survival and proliferation and induction of apoptosis. Furthermore, restoration of mTOR activation by overexpression of a constitutively-active AKT1 (ca-AKT1) form in SKOV3 cells partially mitigated, but did not reverse, CC223-induced cytotoxicity in OvCa cells [171]. Finally, CC223-induced reactive oxygen species (ROS) production in SKOV3 cells and inhibited their in vivo growth in nude mice [171]. Another study demonstrated that a novel PI3K/mTOR dual inhibitor, CMG002, has the ability to overcome the chemo-resistance in OvCa cells to paclitaxel or cisplatin by inhibiting cell proliferation and inducing G1 cell cycle arrest and apoptosis in vitro [174]. In vivo, CMG002 suppressed the growth of OvCa in xenograft in nude mice as a monotherapy, and in combination with paclitaxel or cisplatin. CMG002 markedly inhibited the phosphorylation of mTOR (Ser2448), AKT (Ser473 and Thr308), and ribosomal protein S6 (rpS6, Ser235/236) [174].

## 8. Therapeutic Targeting of PI3K-AKT-mTOR-NFκB in Clinical Trials: Opportunities and Challenges

Due to its multiple downstream effectors, PI3K has become an attractive therapeutic target for OvCa. Several clinical trials that are targeting PI3K in combination with targeting its upstream activators or downstream effectors are ongoing in phase 1 and 2 clinical trials (Table 3). Targeting all Class I PI3K forms by BKM120, or Buparlisib, a dimorpholino pyrimidine derivative [175], is paired with BYL719 (alpelisib), which has targeted phosphatidylinositol 3-kinase α-selective forms [176] in a phase 1 trial. Another inhibitor of all PI3K isoforms, Copanlisib, with the greatest inhibitory activity against PI3Kα and PI3Kδ [177,178], is in a phase 1 trial with MEK162, or binimetinib, that inhibits MEK1/2. Moreover, CUDC-907, an oral small molecule inhibitor that targets both PI3K and HDAC [179] is in a phase 1 trial.

Drugs targeting AKT include AZD5363 and GSK2141795, both of which are potent pan-AKT inhibitors as well as the cell-permeable tricyclic nucleoside, Triciribine (PTX-200) and the allosteric, selective, non-ATP competitive inhibitor, Miransertib (ARQ 092) are all currently in phase 1 clinical trials. MK2206, another allosteric AKT inhibitor, is currently in phase 2. 

Small molecule inhibitors that target mTOR are in phase 2 trials, including sapanisertib, as well as derivatives of macrolide compounds from rapamycin, sirolimus, everolimus, and temsirolimus. Nanoparticle albumin-bound rapamycin, along with MLN0128, an ATP-competitive kinase inhibitor, and AZD2014/vistusertib target mTOR and are in phase 1 trials. 

Interestingly, several therapies have been discovered which target multiple effectors. For example, TAK228 targets PI3K/AKT/mTOR and downregulates inflammatory cytokines IL-6 [180], and is currently in a phase 2 trial. Perifosine targets PI3K/AKT/MTOR as well as NFκB [181] and is currently in phase 1 of trials (Table 3). Encapsulated formulation of curcumin together with RTKI and doxorubicin (IMX-110) is currently in a phase 1/2 trial as it has an advantage of targeting cancer cells with doxorubicin while inhibiting activation of signal transducer and activator of transcription 3 (STAT3) and NF-kB, as well as PI3K/*AKT*/mTOR [182,183] by curcumin, thus inhibiting the evolution of resistance [184,185].

While there are several drugs targeting the PI3K pathway under investigation in clinical trials, it is important to address the challenges in the clinical development of these therapies in OvCa. The success of PI3K inhibitors is inherently limited by several factors. One challenge is the toxicity of PI3K inhibitors, which is that largely dependent upon isozyme specificity; pan-PI3K inhibitors have serious side effects including myelosuppression and transaminitis, while PI3Kα inhibitors result in hyperglycemia and rash [186]. Therefore, one solution to ameliorate drug toxicity would be the development of isozyme specific PI3K inhibitors with lower toxicity profiles. Another challenge includes feedback upregulation of compensatory pathways. Inhibition of PI3K blocks the phosphorylation of FOXO and thus reverses the suppression of RTKs, leading to the depression of ribosomal protein S6 kinase (S6K) and Growth Factor Receptor Bound Protein 10 (GRB10), and hence resulting in the activation of multiple RTKs and PIP3 production [186]. A similar problem is encountered with therapies that aim to induce NFκB inhibition. This inhibition causes an increased activation of the Nucleotide-Binding Oligomerization Domain, Leucine Rich Repeat And Pyrin Domain Containing 3 (NLRP3, NLR family pyrin domain containing 3) inflammasome, which causes a caspase-1 mediated activation of pro-inflammatory cytokines, IL-1β and IL-18 [187]. While this poses a major setback, it can be rectified by using a combination of therapies [186]. A third challenge in using PI3K inhibitors is the enhanced insulin production upon PI3K inhibition; studies have indicated that there is a dose-dependent increase in plasma levels of C-peptide and insulin with PI3K inhibitors and that this insulin secretion may activate insulin receptors and PI3K, thus reducing the overall clinical efficacy of PI3K inhibitors [176]. However, if PI3K inhibitors are used in combination with sodium-glucose costransporter-2 (SGLT2) inhibitors, or if the patient is placed on a ketogenic diet, this effect may be alleviated [186]. 

## 9. Conclusions

Among all gynecologic malignancies, HGSC is the most prevalent and lethal subtype. Women are diagnosed at advanced stages and with limited treatment options. The five-year survival for stage III/IV patients is poor and hovers around 30–40%. Recent studies have indicated that the PI3K/AKT/mTOR, as well as NFκB pathways, are highly mutated and hyper-activated in a majority of OvCa patients, and are associated with aggressive disease, therapeutic resistance, recurrence, and an overall poorer prognosis. A comprehensive investigation of the PI3K/AKT/mTOR and their interconnection with the NFκB pathway will not only expand our understanding of this ruthless disease, but also enhance the development of more effective and targeted therapies in OvCa.

## Figures and Tables

**Table 1:** Summary of mutations in the PI3K-AKT pathway in 13 OvCa cell lines collected from Cancer Cell Line Encyclopedia (CCLE), https://oncokb.org, and [36].

Cell Line	Annotation	PI3K	PTEN	AKT
A2780	Unlikely HGSC/not-specified	*PIK3CA* Missense p.E365K	Deletion	AKT3 Gain
CAOV3	Likely HGSC/adenocarcinoma	*PIK3CA* amplification	Gain	AKT1/2 Gain
IGROV1	Mixed subtype/hypermutated	*PIK3CA* Missense/non-stop p.R38C & p.1069W	p.Y155C Loss of function	
		*PIK3CB* p.A686V		
MCAS	Mucinous cystadenocarcinoma	*PIK3CA* Missense p.H1047R	Gain	
OVMIU	Not-specified	*PIK3CA* Missense p.H1047R		
OVMANA	Clear-cell	*PIK3CA* Missense p.E545V		
OVISE	Clear-cell	*PIK3CA* Missense p.C420R		AKT2 Gain
OC314	Serous	*PIK3CA* Missense p.R108H		
OC316	Low-differentiated adenocarcinoma	*PIK3CA* Missense p.R108H	Heterozygous loss	AKT1 Gain
		*PIK3CG* p.T309M & p.R542W		
EFO27	Mucinous	*PIK3CA* Missense p.H510N		
COV318	Serous/non-specified?	*PIK3CA* Gain/amplification?		AKT1/2/3 Gain
		*PIK3CB* Missense p.W676R & p.V260I		
OVCAR3	Poorly differentiated papillary adenocarcinoma/possibly HGSC	*PIK3CA* AMP		AKT2 amplification/Gain
		*PIK3R1* splice variant c.e14-1 (likely loss of function)		
SKOV3	Moderately well-differentiated adenocarcinoma/mixed subtype	*PIK3CA* Missense p.H1047R	Heterozygous loss	
TOV21G	Clear-cell adenocarcinoma/hyper-mutated	*PIK3CA* Missense p.H1047Y	Gain	
OAW42	Non-specified	*PIK3CA* Missense p.H1047L	Heterozygous loss	AKT2 Gain
FUOV1	Serous			AKT1/2 Gain

**Table 2:** Summary of the tendencies of PI3K/AKT/MTOR/NFκB mutations to co-occur in OvCa (curated from http://www.cbioportal.org).

A	B	Both	Log2 Odds Ratio	*p*-Value	*q*-Value
*PIK3CA*	*PIK3CB*	117	2.813	<0.001	<0.001
*PIK3CA*	*AKT3*	60	1.272	<0.001	<0.001
*PIK3CB*	*MTOR*	21	2.009	<0.001	<0.001
*PTEN*	*AKT1*	23	1.814	<0.001	<0.001
*AKT1*	*IKBKB*	23	1.659	<0.001	<0.001
*PIK3CA*	*PTEN*	75	0.918	<0.001	<0.001
*PIK3R1*	*MTOR*	14	2.132	<0.001	<0.001
*PIK3CA*	*RELA*	37	1.343	<0.001	<0.001
*PIK3CA*	*MTOR*	47	1.109	<0.001	<0.001
*AKT3*	*MTOR*	16	1.808	<0.001	<0.001
*AKT2*	*AKT3*	20	1.576	<0.001	<0.001
*PTEN*	*NFKBIA*	15	1.875	<0.001	<0.001
*PIK3R1*	*IKBKB*	19	1.607	<0.001	<0.001
*AKT1*	*NFKB2*	8	2.582	<0.001	<0.001
*PTEN*	*MTOR*	19	1.484	<0.001	0.001
*AKT2*	*IKBKB*	26	1.247	<0.001	0.001
*IKBKB*	*NFKBIA*	14	1.598	<0.001	0.004
*PIK3CB*	*NFKB1*	8	2.15	0.001	0.005
*PIK3R1*	*AKT1*	11	1.679	0.002	0.006
*PTEN*	*NFKB2*	9	1.929	0.002	0.006
*PIK3CB*	*NFKB2*	8	2.034	0.002	0.007
*PIK3CA*	*AKT2*	57	0.707	0.002	0.008
*AKT3*	*NFKB2*	7	2.08	0.003	0.009
*MTOR*	*NFKB1*	6	2.205	0.004	0.011
*PIK3CB*	*AKT1*	14	1.248	0.005	0.016
*PIK3CB*	*NFKBIA*	10	1.474	0.006	0.019
*AKT2*	*NFKBIA*	10	1.449	0.007	0.021
*PIK3CA*	*NFKBIA*	27	0.897	0.008	0.023
*AKT3*	*NFKB1*	6	1.922	0.008	0.023
*PIK3CB*	*AKT2*	17	0.982	0.012	0.03
*AKT2*	*MTOR*	13	1.12	0.013	0.031
*PIK3CA*	*IKBKB*	68	0.501	0.013	0.031
*NFKB1*	*NFKBIA*	4	2.245	0.014	0.032
*RELA*	*NFKBIA*	6	1.728	0.014	0.032
*RELA*	*IKBKB*	12	1.106	0.018	0.04
*AKT1*	*AKT3*	11	1.129	0.02	0.043
*RELA*	*NFKB1*	4	2.051	0.021	0.045
*RELA*	*NFKB2*	4	1.945	0.026	0.054
*PIK3CA*	*NFKB2*	16	0.96	0.027	0.054
*PIK3CB*	*PTEN*	18	0.772	0.032	0.062
*AKT3*	*RELA*	8	1.176	0.036	0.068

**Table 3:** Current clinical trials targeting the PI3K/AKT/mTOR/NFκB pathway in OvCa.

Drug	Target	Clinical Trial	NCT Trial
CUDC-907	PI3K/HDAC	Phase 1	NCT02307240
Copanlisib	PI3K	Phase 1b	NCT03586661
BKM120/BYL719	PI3K	Phase 1	NCT01623349
MK2206	AKT	Phase 2	NCT01283035
BKM120 + MEK162	PI3K	Phase 1	NCT01363232
AZD5363	AKT	Phase 1	NCT01226316
GSK2141795	AKT	Phase 1	NCT01266954
Triciribine (PTX-200)	AKT	Phase 1	NCT01690468
Miransertib(ARQ 092)	AKT	Phase 1b	NCT02476955
Perifosine	PI3K/ AKT	Phase 1	NCT00431054
Sapanisertib	mTOR	Phase 2	NCT02465060
Everolimus	mTOR	Phase 2	NCT01149434
Nanoparticle albumin-bound rapamycin	mTOR	Phase 1	NCT02646319
Sirolimus	mTOR	Phase 1	NCT02833506
TAK228	PI3K/ AKT/mTOR	Phase 2	NCT03648489
MLN0128	TORC 1/2	Phase 1	NCT02142803
Temsirolimus	mTOR	Phase 2	NCT02093598
AZD2014	mTOR	Phase 1b	NCT02208375
IMX-110	Stat3/NF-kB/poly-tyrosine kinase	Phase 1/2a	NCT03382340
